# The Therapeutic Properties of Bromide of Ammonium

**Published:** 1863-12

**Authors:** Ira Hatch

**Affiliations:** Chicago


ARTICLE XXXV.

### THE THERAPEUTIC PROPERTIES OF BROMIDE OF AMMONIUM.

BY IRA HATCH, M.D., of Chicago.

Communicated to the Chicago Medical Society, Oct. 9, 1863.

The *London Lancet* for April, 1863, contains a notice of the bromide of ammonium.

Dr. GIBB recommends it highly in nervous affections, in diseases of the mucous membranes, and in epilepsy.

It will be recollected that in a communication to this Society, a few months ago, I expressed the opinion that remedies would yet be found that would act far more effectively as sedatives upon the mucous surfaces, than any hitherto known to the profession. If Dr. GIBB'S statement with regard to the bromide of ammonium may be relied on, or if it should be confirmed by further experience, a very important discovery, looking to this end, has already been attained. He says, that "the mucous membrane of the whole body is brought more or less under its control. Trembling, nervousness, and general uneasiness quickly subside under its use. It calms irritation, and allays nervous irritability."

Soon after this account of the bromide fell under my eye, I had some cases which I thought would test the value of the medicine.

One was a case of chronic liver complaint, of long standing, complicated with a diseased condition of the mucous membranes. The patient had had fits of indigestion, vomiting of bile, a sallow skin, and irregularity of the bowels for a long time. She had had chronic bronchial asthma for at least twenty years. She had been subject, from a child, (she is now over forty years old) to distressing turns of sick headache, with vomiting, fainting, and palpitation of the heart. During these paroxysms of headache, the dyspnœa, dizziness, blindness, and numbness were really alarming. Succeeding one of these paroxysms, she had hemiplegia, which lasted several weeks, but finally wore off under the use of counter-irritants to the spine, and the internal use of nux vomica. But it left her nervous, restless, and constantly apprehensive of another attack.

She is a married woman, but has never borne children. The catamenia have always been defective; menstruation sometimes irregular, and at other times painful or altogether wanting.

In order to allay nervous irritation, I gave her bromide of ammonium, in two grain doses, four times a-day. It promptly relieved the nervous irritation, and produced a calm quiet. She was inclined to sleep more than usual.

At present she is taking no medicine regularly. Her health is better apparently than it has been for several years. When she feels particularly nervous, she takes a dose of the bromide, which is not often necessary. I do not anticipate any very lasting benefit. There is too much organic disease to expect it. But the experiment shows the sedative properties of the remedy.

The fungous granulations of the throat and back part of the tongue have very much diminished in size and improved in appearance. But this may not have anything to do with the bromide. I may add here, that she has been greatly benefited by the ext. nux vomica and quinine and iron, to which I mainly attribute the present improved condition of her health.

Another case in which I have used the brom. amm. was a woman thirty-nine years old, fifteen years married, but had never borne children. She is of a nervo-sanguineous temperament, the latter temperament largely prevailing.

A considerable portion of the time since puberty she has been the victim of dysmenorrhœa and leucorrhœa, with occasional turns of dyspepsia. The bowels have generally been constipated. She never has been anæmic or emaciated; but her countenance has generally been indicative of health.

Last fall she became pregnant. In March, the seventh month of her pregnancy, she took hooping-cough, which ultimately produced uterine hemorrhage, followed by pains; and she was delivered of a still born child on the 3d of April last.

The labor, as well might be expected, was tedious and painful. The os uteri was somewhat rigid and excessively sensitive. The pains, though light, distressed her exceedingly. Her recovery was slow and tedious.

She lost her strength and appetite, and became very nervous. She had fits of swooning, evidently of a hysterical character. She laughed and cried in the same breath. She would not be left alone in her room for a moment. Yet there did not seem to be any adequate cause for such nervousness. There was no fever; no tenderness of the abdomen; lochia slight; and no leucorrhœa. Pulse, respiration, and skin natural.

The bowels were constipated, and every evacuation prostrated her wonderfully, and aggravated all her nervous symptoms. No hemorrhoids discoverable. At this time, with no other view than to allay nervous irritability, I prescribed

℞.—Bromide Amm., .................................. ʒij

Syr. Prun. Virg., .................................. ℥iv.

Dose, one teaspoonful every six hours.

The effect was most gratifying. A moderate leucorrhœa immediately made its appearance.

She rested well the first night and ever afterwards. She had no more fainting fits or palpitation. Her recovery, from this time, was steady, uniform, and complete. In June, she went East, where she spent the summer; and I saw no more of her till ten days ago, when I was called to visit her for another complaint.

After her sickness, which was temporary, had subsided, I investigated the old complaint, and found that she had menstruated freely, without pain, and had experienced none of her old troubles, with the exception of a slight leucorrhœa, which, on inspection by the speculum, was found to issue from the neck of the uterus, and was thick, tenacious, and transparent.

She told me in the presence of a homœopathic lady, that she had a remedy in the bromide of ammonium that would control the pain and nervousness which had tormented her so long, and for which she had never before found any relief, except by opium; and that destroyed her appetite, and generally made her sick for a week afterwards. She is an intelligent lady, and I think her testimony is worth something. She is one of the kind, too, who never have had much faith in medicines, being homœopathically inclined.

The medicine seemed to act, as a direct sedative, upon the mucous membrane of the cervex uteri, much in the same manner as cleavers act upon an irritated urethra, or as buchu acts upon the bladder. It allayed the local irritation, and the constitutional symptoms disappeared.

The patient had been tormented for years with painful menstruation and uterine leucorrhœa. The primary difficulty was located in the neck of the uterus. Dr. TYLER SMITH says, (what we all know to be true) that the mucous membrane of the upper portion of the neck is sometimes remarkably sensitive; and is nearly as intimately connected with the mental emotions as the lachrymal gland.

This accounts for leucorrhœa from mental excitement. The hysteria, palpitation of the heart, and other nervous symptoms of our patient were the result of the excessive irritability of the upper portion of the neck or inner sphincter, usually called the os internum. In nervous irritable women, rendered so by uterine troubles, is not the point of irritation situated in this portion of the cervix uteri? This portion is highly organized, and is always sensitive, even in the healthy woman; made so, undoubtedly, to guard the sacred enclosure within.

A highly organized, sensitive part becomes doubly so whenever the action of such part becomes diseased. The os internum sometimes becomes very irritable in pregnancy; causing obstinate vomiting, and other distressing nervous symptoms. It would seem, sometimes, as if this part of the neck were the centre of nervous sympathy in the female. Not only the stomach but the uterus itself and the ovaries are powerfully influenced by any disturbance of this part. It is well known that a very slight but continued artificial irritation of the os internum will cause abortion. Nothing more is needed than to pass through it a smooth elastic tube, and let it remain till pains come on. In amenorrhœa, depending upon certain local causes, the tube is a sovereign remedy. In vicarious menstruation this course is certain to restore the natural mentrual flow.

Some years ago, I saw a striking exemplification of this truth, in a lady long afflicted with vicarious menstruation. Each menstrual period was preceded by the most distressing symptoms of congestion of the lungs; followed by a true catamenial secretion from the lungs, (unless prevented by treatment.) The patient had been many times copiously bled, and subjected to a variety of treatment. She was pale, thin, nervous, and hysterical; thus verifying the assertion of Dr. PREECH, of Paris, that "in all cases of vicarious menstruation which had been carefully observed, antecedents either of hysteria, or of an exaggerated sensibility, have been noticed."

In this condition, she put herself under the care of Dr. KIBBEE, of Springfield, Mass. He treated the case by introducing the tube every month; anticipating the menstrual period, generally, a day or two.

She always menstruated, per "*vias naturales*," within forty-eight hours after the introduction of the tube. She eventually recovered, and is now well and hearty.

I introduce this case to show the effect of irritation of the os internum, when produced artificially. Does not menorrhagia sometimes arise from irritation, owing to a morbid condition of the os internum? Do not abortions often arise from the same cause? Does not dysmenorrhœa almost always arise from an excessive sensitiveness, and consequent spasmodic contractility of the os internum, which is in fact a sphincter? Do not puerperal convulsions, especially those of a hysterical character, sometimes arise from the same cause?

Judging from the effect produced by artificial irritation of the os internum, we may infer that like effects would ensue from a morbid condition or irritation of this part. Any remedy that will allay irritation in this centre of sympathy in the female, would be of inestimable value. I do not pretend to say, that bromide of ammonium will do it. It requires further trial. I *think* it applicable in all cases of nervous females, where the nervousness arises from uterine irritation. It may extend to all irritations of mucous surfaces.

